# Effects of cactus (*Opuntia ficus-indica*) mucilage on boar sperm cryosurvival

**DOI:** 10.1590/1984-3143-AR2024-0004

**Published:** 2025-06-30

**Authors:** Juan Antonio Ramírez-Chequer, Marco Antonio Lopez-Carlos, Carlos Fernando Arechiga-Flores, Pedro Hernandez-Briano, Carlos Aurelio Medina-Flores, Fabiola Mendez-Llorente

**Affiliations:** 1 Unidad Académica de Medicina Veterinaria y Zootecnia, Universidad Autónoma de Zacatecas, General Enrique Estrada, Zacatecas, Mexico.

**Keywords:** antioxidant, freezing extender, plant extracts, swine

## Abstract

This study aimed to evaluate the effect of three inclusion levels of *Opuntia ficus-indica* mucilage (OFM) to the lactose-egg yolk extender on the quality of frozen-thawed boar semen. Semen samples (n=22) were split into four OFM treatments: 0.0 (control), 3.3, 6.7, or 10.0% OFM. The semen was packaged in 0.5 mL straws and frozen using conventional methods. The sperm motility, viability, membrane integrity (HOST), and morphology (intact acrosome and normal sperm) characteristics were evaluated immediately post-thaw. Additionally, sperm were incubated at 37 ºC during 3.5 h and monitored hourly in a termoresistance test (TRT) to assess sperm motility, viability, and membrane integrity, The study showed that adding OFM to the freezing extender significantly improved (P < 0.05) sperm quality characteristics, without affecting (P > 0.05) acrosome integrity or sperm abnormalities. Furthermore, the OFM addition improved (P < 0.05) the sperm viability and membrane integrity for up to 3.5 h of incubation. Results indicate that OFM can be added to the extender at a 6.7% OFM inclusion level to improve the quality of cryopreserved boar sperm.

## Introduction

In recent decades, the progress in pig production has been supported by reproductive biotechnologies such as artificial insemination with liquid or frozen preserved semen (Mcnamara and Knox, 2013). Frozen semen offers the advantages of unlimited storage time and availability over long distances, facilitating international trade (Waberski et al., 2019). This biotechnology remains one of the primary techniques for propagating genetic material in many animal species (Smith et al., 2018). However, less than 1% of swine artificial inseminations worldwide use frozen semen (Yeste, 2017).

The most commonly used freezing diluent for this species is the lactose egg yolk diluent (LEY), as it consistently produces satisfactory results (Holt, 2000). This diluent (LEY), combined with glycerol as a cryoprotectant (Maes et al., 2011; Pezo et al., 2019), helps maintain osmotic pressure, protects against cold shock, and stabilizes the sperm membranes during the freezing-thawing process (Oldenhof et al., 2021). However, boar sperm are particularly vulnerable to the stresses of cryopreservation, such as the production of reactive oxygen species (ROS) that can compromise sperm functionality (Rodríguez-Martínez and Wallgren, 2010; Yeste, 2017; Chanapiwat and Kaeoket, 2020). Current research in this field is focused on enhancing post-thaw results, either by adjusting existing freezing protocols or by adding various additives and antioxidants to the freezing diluent to protect the sperm and maintain quality parameters after the freezing-thawing process (Pezo et al., 2019; Chanapiwat and Kaeoket, 2020; Yánez-Ortiz et al., 2022).

The *Opuntia* genus, also known as nopal cactus, consists of dicotyledonous plants in the *Cactaceae* family. These plants are indigenous to tropical and subtropical regions, thriving primarily in arid and semi-arid climates (Labra et al., 2003). Its geographical distribution includes North and South America, the Mediterranean Basin, the Middle East, South Africa, India, Thailand, and Australia (Anderson, 2001). There are more than 200 species of *Opuntia*, 20 of which have been cultivated to varying degrees of domestication (Chávez-Moreno et al., 2009). The most widely cultivated *Opuntia* species is *O. ficus-indica*. It is cultivated primarily as animal fodder (Dubeux et al., 2021) but it is also used as a source of food and traditional medicine for humans (Hernández-Becerra et al., 2022). In addition, *O. ficus-indica* is a valuable source of beneficial compounds for the chemical and pharmaceutical industries (Aruwa et al., 2018). The *Opuntia* species has many potential uses in nutrition, medicine, and pharmaceuticals with beneficial effects such as neuroprotection, anti-inflammation, antioxidant, antiulcer, antimicrobial, antiviral and skin regeneration (Trachtenberg and Mayer, 1981; El-Mostafa et al., 2014; Castañeda-Arriaga et al., 2021).

Several studies (Malo et al., 2011, 2012; Chanapiwat and Kaeoket, 2015; Gale et al., 2015; Luño et al., 2015; Montont et al., 2015; Shen et al., 2015; Ratchamak et al., 2020; Ros-Santaella and Pintus, 2021; Lee and Lee, 2023) have reported the use of plant extracts for their antioxidant effects in protecting boar semen during cryopreservation. Other studies (Gomez-Fernandez et al., 2012; Qian et al., 2016; Pezo et al., 2021) have reported the use of chemical compounds with cryoprotective properties, showing positive results in most cases. *Opuntia ficus-indica* is known for its significant antioxidant properties, attributed to its abundance of phenolic compounds, vitamin C, and key amino acids such as glutamine and leucine (El-Mostafa et al., 2014; Castañeda-Arriaga et al., 2021). These properties could potentially reduce the oxidative damage induced to sperm cells during the cryopreservation process.

In addition, *Opuntia* species can survive long periods of drought by storing water in the parenchyma of their cladodes in the form of mucilage (Nuzhyna et al., 2018). The mucilage found in *O. ficus-indica* is a hydrocolloid composed mainly of sugars, including arabinose, galactose, rhamnose, xylose, and galacturonic acid in polymeric form, together with various other pectic compounds with thickening and water molecule retention properties (Trachtenberg and Mayer, 1981; Stintzing and Carle, 2005). The large sugars cannot move through the cell walls, and therefore help prevent excessive water movement into cells by creating greater osmotic pressure. This makes it harder for ice to form inside the cells, which could help protect sperm when it is frozen (Fuller, 2004; Gómez-Fernández et al., 2012).

In this regard, previous research has indicated that adding acetone extract (Allai et al., 2017), ethanolic extract (Allai et al., 2023), or aqueous extract (Hussein Banana et al., 2021) of *O. ficus-indica* cladodes in extenders for liquid storage (5 °C) of ram semen for 72 h improves sperm motility, viability, or morphology because it reduces DNA fragmentation and lipid peroxidation. However, the extraction methods described by the authors involve complex mucilage extraction techniques that involves costly reagents, solvents, or specialized equipment. In addition, no previous studies have been reported about the use of OFM for freezing semen of any species. Due to the remarkable susceptibility of porcine species to stress during freezing-thawing process, it may be valuable to explore the potential osmotic and antioxidant benefits of fresh plant-derived OFM in boar sperm cryopreservation.

Therefore, this study aimed to evaluate the protective effects of OFM and its optimal concentration to preserve the quality of boar sperm after cryopreservation. We hypothesize that the OFM inclusion in the freezing extender improves the motility and viability parameters of boar sperm subjected to the freezing-thawing process.

## Methods

The study was carried out in the Swine Research and Teaching Farm of the Academic Unit of Veterinary Medicine and Zootechnics of the Autonomous University of Zacatecas, Mexico. The research protocols, animal care and handling procedures were in accordance with the Official Mexican Standard NOM-062-ZOO-1999 (México, 2001), about technical specifications for the production, care and use of laboratory animals, as well as the ARRIVE (Animal Research: Reporting of In Vivo Experiments) guidelines and was performed following the UK Animals (Scientific Procedures) Act 1986 and associated guidelines, EU Directive 2010/63/EU for Experiments with animals. All procedures were approved by the Bioethics and Animal Welfare Committee UAMVZ-UAZ of Autonomous University of Zacatecas (2022/01).

### Procedures for obtaining mucilage

Cladodes were gathered from *O. ficus-indica* which had been planted four years earlier in an area that had not been treated with any chemical substances or fertilizers, in the Academic Unit of Veterinary Medicine and Zootechnics at the Autonomous University of Zacatecas. The harvest was conducted on the same day the cladodes were used, between September and November. Spines were removed and the cladodes were subsequently washed. Subsequently, they were washed with distilled water to remove any surface impurities. The rind was stripped off, leaving only the inner pulp. The fresh pulp was squeezed by applying manual pressure through a sterilized gauze sieve to remove the plant tissue and collect the viscous portion, known as mucilage. The mucilage was placed into 50 mL conical tubes and centrifuged at 1,200 × g for 15 min. The supernatant mucilage was decanted into clean tubes to remove impurities and remaining plant tissue from the bottom of the tube. The resulting mucilage, with a pH of 5.8, was adjusted to a more neutral pH (close to 7.0) by adding 5.4 mg/mL of NaHCO_3_. It was used immediately in the treatments or refrigerated at 5 °C for later use.

### Animals and general management

Four healthy boars, aged 13 ± 0.5 months (1 York and 3 Pietrain × Duroc × Landrace), regularly used for semen collection, were chosen for the trial. Before the start of the experiment, four ejaculates were collected from each boar, with a one-week interval between collections. These ejaculates were then frozen using the standard cryopreservation procedure to evaluate their suitability as “good” freezers. This was done to ensure consistency in the cryosurvival of boar sperm (Roca et al., 2006). During the previous semen collections, the average post-thaw total motility was 38.2% (range 29.4-43.3%) and progressive motility was 23.9% (range 16.3-30.4%). The animals were housed in individual pens with ad libitum access of clean water and were fed twice daily with a balanced feed containing 16% crude protein (CP).

### Semen collection and handling

Semen samples (n = 22) were collected from each boar, one week apart. Two boars (1 York and 1 Pietrain × Duroc × Landrace) provided 8 samples each, while the other two boars (Pietrain × Duroc × Landrace) provided 3 samples each. Semen samples were collected using the gloved hand technique, taking care to collect the sperm-rich fraction in a semen collection container with a plastic bag and a filter (Minitube, Germany). The semen was transported to the laboratory in a polystyrene box, ensuring it was maintained at an average temperature of 35 °C until routine evaluation within 30 min of collection (Pezo et al., 2019; Oldenhof et al., 2021).

At the laboratory, semen samples were assessed for macroscopic characteristics such as color, volume, and odor, as well as microscopic characteristics such as concentration, motility, viability, morphology, and HOST reaction (Evans and Maxwell, 1987; Maes et al., 2011). The evaluation was conducted using a Leica DM2500 microscope (Leica Microsystems). The hemocytometer method was used to measure the sperm concentration (×10^6^ cells/mL). Sperm motility was analyzed subjectively by placing a one drop (10 μL) of semen on a slide warmed at 37 °C and covered with a clean coverslip (24 × 24 × 1.5 mm) under a bright-field objective at 400× (Broekhuijse et al., 2012). Viability was counted in an eosin-nigrosin stained smear and evaluated using bright-field objectives at 400 and 1000× respectively and reported as percentage of live sperm (Kondracki et al., 2017). Sperm abnormalities were assessed in smears stained with the Diff-Quik staining method (Agarwal et al., 2022). The smears were evaluated under the bright field objective at 1,000× in immersion oil, counting at least 200 sperm cells. Normal and abnormal sperm were both recorded. The results were expressed as the percentage of normal sperm. The HOST was performed by the wet chamber technique. A sample of 50 µL of raw boar semen was mixed with 1 mL of a fructose-sodium citrate solution (100-150 mOsm/L) and gently incubated for 30 minutes at 37 °C. Following incubation, 10 µL of the semen and hypoosmotic solution mixture was placed on a slide and covered with a coverslip. Under a phase-contrast microscope at 400× magnification, at least 200 sperm cells were counted across a minimum of five fields. Sperm cells that exhibited swelling in the tail were categorized as having intact membranes. The percentage of sperm displaying bent and curled tails in the morphology analysis was subtracted from the total number of sperm responsive to the hypoosmotic swelling test (HOST).

Only ejaculates with a concentration of ≥ 200×10^6^ cells/mL, ≥ 80% normal morphology, ≥ 80% motility, and ≥ 85% viability were used to proceed with semen processing (Table 1). The semen was diluted 1:1 v/v (Jovičić et al., 2020) in a commercial extender (Androhep, Minitube, Germany) at 35 °C and allowed to cool for 1 h at room temperature (23 °C). The diluted semen was cooled from 23 °C to 17 °C over 1.5 h inside a temperature-controlled refrigerated cabinet. It was then kept at 17 °C for approximately 24 h before proceeding with the freezing procedure (Torres et al., 2019). The initial sperm concentration on ejaculates was 667.2 cells x 10^6^/mL average, therefore when making the 1:1 v/v (Jovičić et al., 2020) dilution the average concentration was 333.6 cells x 10^6^/mL (Table 1).

**Table 1:** Characteristics of raw boar semen (n = 22 S.D. = standard deviation, C.V. = coefficient of variation).

**Characteristic**	**Mean**	**S.D.**	**C.V. (%)**
Volume (mL)	199.8	54.3	27.2
Concentration (cells x 10^6^/mL)	667.2	284.3	42.6
Temperature (°C)	33.8	1.4	4.1
pH	7.1	0.1	1.4
Total motility (%)	89.4	3.7	4.2
Viability (%)	92.5	2.6	2.9
Normal morphology (%)	87.4	4.1	4.7
Hyposmotic test reaction (%)	76.7	5.6	7.3

### Freeze-thaw procedure and description of treatments

The composition and characteristics of pre-freezing extender A and freezing extender B are shown in Table 2. The freezing extender B had four levels of OFM inclusion (treatments). The final pH was measured in the extenders each time they were prepared. Osmolarity was calculated for each extender (without glycerol and LSS) as described by Rasouli (2016) with the formulae mOsm/L = Σ ([Ii × Oi] / total volume) × 1000; where Ii = volume of each ingredient in mL, Oi = osmolarity of each ingredient in mOsm/mL. The calculations were based on published values. LEY extender = 330 mOsm/L (De Mercado et al., 2020; Tomás et al., 2011). OFM = 260 mOsm/L (Goldstein et al., 1991; Goldstein and Nobel, 1994) plus 128.6 mOsm/L from 5.4 mg/mL NaHCO_3_ addition (Senewiratne et al., 2024) resulted in total OFM osmolarity of 388.6 mOsm/L.

**Table 2:** Composition of freezing extenders containing *Opuntia ficus-indica* mucilage (OFM).

**Ingredients for 100 mL 1**	**Freezing extender A**	**Freezing extenders B (treatments) 2**			
		**Control**	**3.3**	**6.7**	**10.0**
LEY extender, mL3	100	91	81	71	61
OFM, mL4	0	0	10	20	30
Glycerol, mL	0	9	9	9	9
Sodium lauryl sulfate (LSS), mg	0	360	360	360	360
pH5	6.80	6.81	6.85	6.82	6.83
Osmolarity, mOsm/L6	330.0	330.0	329.8	329.6	329.4

^1^All extenders contained tylosin (50 μg/mL), gentamicin (250 μg/mL), and lincomycin (150 μg/mL); ^2^Sperm cells were initially diluted in freezing extender A to obtain 1.5 × 10^9^ sperm/mL. After equilibration period at 5 °C, the freezing extenders B (treatments) were added at 1/3 of the diluted semen volume to achieve the desired final concentration of 1 × 10^9^ sperm/mL, 3% glycerol, 1.2 mg/mL LSS. The final OFM concentration was 0.0, 3.3, 6.7, and 10.0% for the respective treatments; ^3^LEY (lactose-egg yolk) extender was composed of 11% lactose solution (v/v) + 20% egg yolk (v/v); ^4^OFM was added with 5.4 mg/mL of NaHCO_3_ to adjust pH nearest to 7.0; ^5^Mean pH values. The D.S. was on average ± 0.06; ^6^Osmolarity values (without glycerol and LSS) were calculated as described by Rasouli (2016) with the formulae mOsm/L = Σ ([Ii × Oi] / total volume) × 1000; where Ii = volume of each ingredient in mL, Oi = osmolarity of each ingredient in mOsm/mL. The calculations were based on published values. LEY extender = 330 mOsm/L (de Mercado et al., 2020; Tomás et al., 2011). OFM = 260 mOsm/L (Goldstein et al., 1991; Goldstein and Nobel, 1994) plus 128.6 mOsm/L from 5.4 mg/mL NaHCO_3_ addition (Senewiratne et al., 2024) resulted in total OFM osmolarity of 388.6 mOsm/L.

The cryopreservation process followed the methods outlined by Kim et al. (2020) and Suwimonteerabutr et al. (2021). Briefly, the semen samples were centrifuged in 50 mL conical tubes at 800 × g for 10 min. After removing the supernatant, the sperm pellet was resuspended in LEY pre-freezing extender A to achieve a concentration of 1.5 × 10^9^ sperm/mL. The tubes containing the diluted semen were gradually cooled by placing them in a beaker with a water jacket at 17 °C and placing it in a commercial temperature-controlled refrigerator for 1.5 hours until it reached 5 °C, after which it was stored for another 2 hours. After the storage period, semen diluted with pre-freezing extender A was split into four portions and randomly assigned to one of four treatments (freezing extenders B + OFM). The extender B corresponding to each treatment was added in a 1:2 proportion (freezing extender B: semen samples diluted with pre-freezing extender A) to achieve the desired final concentration of sperm cells (1 × 10^9^ sperm/mL) and final OFM inclusion level (0, 3.3, 6.7, and 10.0%).

The semen sample remained for 1 h at 5 °C. After that, packaged in 0.5 mL plastic straws (IMV Technologies, L'Aigle, Normandy, France) and sealed with polyvinyl alcohol powder. The straws were suspended on a rack 4 cm above the surface of liquid nitrogen (-120 °C) for 12-15 min. Then, they were directly immersed in liquid nitrogen and stored in a cryogenic thermos. To thaw the straws, they were placed in a water bath at 52 °C for 12 s and then at 37 °C for 60 s (Chanapiwat and Kaeoket, 2020; He et al., 2020). The samples were then placed in a 15 mL conical tube, diluted in Androhep (1:4, v/v), and evaluated at 37 °C for 30 min for sperm motility and viability traits.

Following a 7-d period, two straws per treatment and sampling period were thawed to assess sperm quality. Furthermore, sperm was subjected to an incubation of 37 °C for periodic monitoring at 0.5, 1.5, 2.5, and 3.5 h to detect any decline in motility and viability over a set time frame.

### Post-thawing sperm quality evaluation

The assessment of motility was performed using a CASA system (Computer Assisted Sperm Analysis, Minitube, Germany) and Sperm Vision software version 1.15.2 (Minitube of America - MOFA®, Verona, WI, USA) connected to a Zeiss Axiolab AX10 (Carl Zeiss MicroImaging GmbH, Göttingen, Germany) phase-contrast microscope equipped with a heating plate with preset temperature at 37 °C. Disposable counting chambers, 20 µm, (Catalogue no. 12050/0220, Minitüb GmbH, Tiefenbach, Germany) were preheated to 37 °C and filled with semen samples (3 µl) according to manufacturer's specifications. Each evaluation involved 8 microscopic fields and counting an average of 1,967 ± 79 sperm cells.

Software settings were as follows: a frame rate of 60 Hz with 45 frames captured; a minimum contrast of 4, and a minimum cell size of 7 pixels. Total motility included any sperm showing movement, while progressive motility refers to sperm that swim mostly in a straight line with an average speed (VAP) of at least 25 µm/s and a straightness coefficient (STR) of at least 30% (Broekhuijse et al., 2012).

To determine the efficiency of the cryopreservation process, the motility recovery rate was calculated. Total and progressive motility recovery rates were obtained by dividing the post-thaw motility value by the pre-frozen motility (after adding the freezing extender A) and expressing the result as a percentage (Ricci et al., 2009; Zhang et al., 2012).

The evaluation of viability, morphology and HOST reaction was carried out using the same techniques as previously outlined for ejaculates (Vazquez, et al., 1997; Pérez-Llano et al., 2001). Acrosome integrity was assessed in smears stained with Diff-Quik staining method (Agarwal et al., 2022). The smears were evaluated under the bright-field objective at 1000× in immersion oil, counting at least 200 sperm cells. An acrosome was considered intact if it was smooth and undamaged.

## Statistical analysis

The sample size was verified through a statistical power test, using the means and standard deviations of data previously obtained in our laboratory with frozen boar semen. The sample size n = 22 ejaculates, obtained a power greater of 0.95. Data were analyzed using SAS OnDemand for Academics software. Normality and homogeneity of variance were confirmed using the PROC UNIVARIATE procedure of SAS (SAS University) before proceeding with statistical analyses. When necessary, data was transformed using square root or arcsine transformation. The PROC GLM procedure was used for the analysis of variance. Data were analyzed as a randomized complete block design, with boar as the blocking variable. For sequential measurements made in the same experimental unit (incubation periods), variables were analyzed as repeated measures. To establish differences and make comparisons between treatment means, the PDIFF instruction with Tukey adjustment was used (SAS Institute, 2018). A value of α less than or equal to 0.05 was considered significant for establishing statistical differences.

## Results

As shown in Table 3, the inclusion of 6.7 or 10.0% OFM in the freezing extender significantly (p < 0.05) improved most motility percentages (total motility, and recovery rates) compared to the control treatment. The greatest (p < 0.05) progressive motility was reached at 6.7% OFM inclusion. However, the addition of only 3.3% OFM was found to be insufficient to improve motility percentages consistently. The study showed that the addition of OFM at any inclusion level (3.3, 6.7, and 10.0%) increased (p < 0.05) the percentage of viability compared to the control treatment. The inclusion of 6.7 or 10.0% OFM to the freezing extender improved (p < 0.05) the sperm reacted to HOST, but 3.3% OFM addition was similar (p > 0.05) to the control treatment. Furthermore, OFM addition to the freezing extender did not affect morphological variables such as acrosomal integrity and normal sperm (p > 0.05).

**Table 3:** Effect of adding three levels of Opuntia ficus-indica mucilage (OFM) to boar sperm freezing extender on motility, viability, and normal morphology immediately after thawing.

	***Opuntia ficus-indica* 1**					
Characteristic (%)	0.0	3.3	6.7	10.0	SEM	P <
Total motility	40.2^b^	42.6^b^	49.6^a^	49.2^a^	0.9	<0.001
Total motility recovery rate	48.2^b^	50.7^b^	58.9^a^	58.7^a^	1.7	<0.001
Progressive motility	25.5^c^	30.3^b^	34.7^a^	32.1^b^	1.0	<0.001
Progressive motility recovery rate	44.0^b^	51.4^ab^	57.5^a^	54.9^a^	2.4	0.001
Viability	51.5^c^	56.3^b^	61.0^a^	56.9^b^	0.8	<0.001
Hypoosmotic test reaction	39.2^b^	45.0^a^	46.3^a^	46.9^a^	0.9	<0.001
Intact acrosome	77.7	74.9	81.1	78.7	1.7	0.079
Normal morphology	85.8	81.1	82.1	81.6	1.0	0.074

^1^Treatments consisted of *Opuntia ficus-indica* mucilage (OFM) added to the lactose-egg yolk freezing extender at final concentrations of 0.0, 3.3, 6.7, and 10.0%. ^a,b^Means with different letters in the same row are statistically different (p < 0.05). SEM = standard error of the mean.

As shown in Table 4, the inclusion of OFM in the freezing extender had a significant effect (p < 0.001) on motility characteristics. The inclusion of 6.7 or 10.0% OFM in the freezing extender led to a significant improvement (p < 0.05) in sperm motility, including total motility, progressive motility and recovery rates, as compared to the Control treatment. However, there were no effects on either the incubation time or the interaction treatment × time (p > 0.05).

**Table 4:** Effect of incubation time at 35 °C after thawing on motility of boar semen added with *Opuntia ficus-indica* mucilage (OFM) in the freezing extender.

**Characteristic (%) / incubation time (h)**	***Opuntia ficus-indica* 1**				**Means Time**	**SEM**	**Trat**	**Time**	**Trat × Time**
	**0.0**	**3.3**	**6.7**	**10.0**					
Total motility						1.5	<0.001	0.258	0.291
0.5	41.4	42.3	51.0	50.5	46.3				
1.5	41.0	42.2	51.0	51.1	46.3				
2.5	38.9	42.9	48.4	47.8	44.5				
3.5	39.5	42.7	48.0	47.4	44.4				
Means Trat	40.2^b^	42.5^b^	49.6^a^	49.2^a^					
Total motility recovery rate						2.1	<0.001	0.354	0.966
0.5	49.5	50.1	60.3	60.2	55.0				
1.5	48.9	50.3	60.4	60.5	55.0				
2.5	46.9	51.5	57.4	57.4	53.3				
3.5	46.6	50.9	57.4	56.4	52.8				
Means Trat	48.0^b^	50.7^b^	58.9^a^	58.6^a^					
Progressive motility						1.5	0.001	0.262	0.3253
0.5	25.3	28.6	34.5	34.5	30.7				
1.5	25.5	30.6	35.8	32.2	31.0				
2.5	25.6	29.3	34.4	33.2	30.6				
3.5	25.5	28.7	34.0	28.5	29.2				
Means Trat	25.5^c^	29.3^b^	34.7^a^	32.1^ab^					
Progressive motility recovery rate						3.5	<0.001	0.421	0.479
0.5	42.9	47.2	56.5	60.2	51.7				
1.5	42.4	52.6	59.8	59.5	53.6				
2.5	45.1	55.7	57.5	58.2	54.1				
3.5	45.4	49.9	56.0	48.8	50.0				
Means Trat	44.0^b^	51.4^ab^	57.5^a^	56.7^a^					

^1^Treatments consisted of *Opuntia ficus-indica* mucilage (OFM) added to the lactose-egg yolk freezing extender at final concentrations of 0.0, 3.3, 6.7, and 10.0%. ^a,b^Means with different letters in the same row are statistically different (p < 0.05). SEM = standard error of the mean, Trat = treatment effect, Time = incubation time effect

The data in Table 5 shows a significant treatment effect (p < 0.001) on the percentages of viability and sperm with a positive reaction to HOST. The inclusion of 6.7% OFM in the freezing extender significantly enhances (p < 0.05) the proportion of viability. Additionally, the addition of OFM at any inclusion level (3.3%, 6.6%, or 10.0%) improved the proportion of sperm reacted to HOST. After 3.5 hours of incubation, the percentages of viable sperm and sperm reacted to HOST drop significantly (p < 0.001). No significant treatment × time interaction was detected (p > 0.05).

**Table 5:** Effect of incubation time at 35 °C after thawing on viability and HOST reaction of boar semen added with *Opuntia ficus-indica* mucilage (OFM) in the freezing extender.

**Characteristic (%) / incubation time (h)**	***Opuntia ficus-indica* 1**				**Means Time**	**SEM**	**Trat**	**Time**	**Trat × Time**
	**0.0**	**3.3**	**6.7**	**10.0**					
Viability									
0.5	58.4	61.6	65.7	62.1	62.0^x^	1.4	<0.001	<0.001	0.6353
1.5	54.3	58.8	63.8	58.7	58.9^x^				
2.5	48.9	56.6	59.6	54.8	55.0^y^				
3.5	44.4	48.3	54.9	51.9	49.9^z^				
Means Trat	51.5^c^	56.3^b^	61.0^a^	56.9^b^					
Hypoosmotic test reaction									
0.5	43.7	46.5	49.0	49.2	47.1^x^	1.7	<0.001	<0.001	0.776
1.5	42.1	47.0	48.8	47.8	46.4^x^				
2.5	36.9	44.5	46.2	47.1	43.7^xy^				
3.5	34.0	42.1	41.1	43.6	40.2^y^				
Means Trat	39.2^b^	45.0^a^	46.3^a^	46.9^a^					

^1^Treatments consisted of *Opuntia ficus-indica* mucilage (OFM) added to the lactose-egg yolk freezing extender at final concentrations of 0.0, 3.3, 6.7, and 10.0%. ^a,b^Means with different letters in the same row are statistically different (p < 0.05). ^x,y,z^Means with different letters in the same column are statistically different (p < 0.05). SEM = standard error of the mean; Trat = treatment effect; Time = incubation time effect.

## Discussion

Cryopreservation causes a decrease in sperm quality, reducing their motility, viability, and fertilizing capacity. This occurs because the process causes structural changes in the sperm due to the osmotic stress, the formation of intracellular ice, and the excessive generation of ROS during freezing and thawing (Yeste, 2017; Yanez-Ortiz, et al., 2022). In cattle, cryopreservation can reduce sperm motility and viability by up to 50%. However, in pigs, the reduction in sperm motility and viability is even more remarkable, at approximately 60% (Yanez-Ortiz et al., 2022), indicating a higher sensitivity to structural damage (Roca et al., 2006). In the present study, we observed a decrease in sperm motility because of the cryopreservation process. These findings align with previous research that utilized the LEY freezing extender for boar sperm cryopreservation (Yeste et al., 2013; Ratchamak et al., 2020; Brito et al., 2021). Yet, this extender is commonly utilized for cryopreserving boar sperm due to its consistent and satisfactory results (Rodríguez-Martínez and Wallgren, 2010; Yeste, 2017; Oldenhof et al., 2021).

However, in this study, it was found that adding 6.7% OFM to the LEY extender resulted in significant improvements (p < 0.05) in total motility (23.4%), total motility recovery rate (22.2%), progressive motility (36.1%), progressive motility recovery (30.7%), viability (18.4%), and reaction to the HOST (18.1%) when compared to the Control group.

Nopal mucilage is a viscous substance found in the cladodes, skin, and fruit pulp (Trachtenberg and Mayer, 1981). It is composed of arabinose, galactose, rhamnose, xylose, uronic acid, and galacturonic acid, as well as simple sugars (glucose and fructose), amino acids, polyphenols, vitamins, and polyunsaturated fatty acids with thickening and water molecule retention properties (Stintzing and Carle, 2005; Procacci et al., 2021). In this sense, it has been reported that the sugars present in the freezing extender can act as cryoprotectants by forming complexes with membrane proteins and lipids (Salamon and Maxwell, 2000; Fuller, 2004). Additionally, high molecular weight sugars cannot diffuse across the plasma membrane. This helps to prevent excessive water movement into cells by creating greater osmotic pressure that causes cellular dehydration and reduces the likelihood of intracellular ice formation (Fuller, 2004; Gómez-Fernández et al., 2012), which could contribute to the protection of sperm during cryopreservation. This could be a possible explanation for the observed improvement in the sperm quality parameters in this study.

On the other hand, Pezo et al. (2021) mention that the ROS produced during the freezing-thawing process damages the sperm's physical structure, reducing the quality and fertility of sperm. Because of their proven effectiveness and low toxicity, the addition of natural antioxidants derived from plants on extenders for sperm storage has been tested in numerous research (Aruwa et al., 2018). The OFM has important antioxidant properties since it contains high amounts of phenolic compounds, vitamin C, and amino acids such as glutamine and leucine (El-Mostafa et al., 2014; Castañeda-Arriaga et al., 2021). These antioxidant compounds have been previously tested either individually or in combination, demonstrating their effectiveness in protecting boar sperm by minimizing the damaging effect of ROS during cryopreservation (Chanapiwat and Kaeoket, 2020; Pezo et al., 2021; Ros-Santaella and Pintus, 2021). In the current study, it was observed that the addition of OFM to the freezing diluent significantly enhanced the motility and survival of post-thawing sperm cells, probably due to the presence of antioxidants in OFM.

Previous studies did not report the inclusion of fresh OFM to sperm extenders. However, some authors argue that the 1% inclusion of seeds extract (Allai et al., 2017) or cladodes extracts of *O. ficus-indica* to the extenders for the liquid storage (5 °C) of ram sperm improved (p < 0.05) sperm motility, viability, and morphology (Hussein Banana et al., 2021; Ros-Santaella and Pintus, 2021; Allai et al., 2023). These effects were commonly attributed to the antioxidant presence in the *O. ficus-indica* extracts that led to a reduction (p < 0.05) in lipid peroxidation levels and DNA fragmentation. Furthermore, in a study conducted by Hfaiedh et al. (2014), the authors provided OFM orally in adult male rats with induced oxidative stress, finding an increased sperm concentration and motility, as well as increased enzymatic activity (SOD, CAT, and GPx).

In line with the positive findings regarding the sperm viability and motility of the present study, Contino et al. (2023) showcased the antioxidant properties of extracts from *Opuntia* fruits, obtained from two *Opuntia* species (*O. ficus-indica* and *O. dillenii*), when added to the freezing extender of human sperm. The contents of *Opuntia* fruits include betalains, flavonoids, and vitamins, which act as free radical scavengers due to their chemical ring structures. These structures are capable of stabilizing dispersed electrons, blocking the activation of a chain reaction, and preventing the passage of electrons (Ďuračková, 2014).

Conversely, Meamar et al. (2012) tested the effects of *Opuntia* fruit extracts (0.5%) and resveratrol (15 μM) addition in the extender for human sperm cryopreservation. They observed that the addition of both extracts reduced (p < 0.05) the DNA fragmentation but did not affect (p > 0.05) the motility or viability parameters. The authors considered that the antioxidant content in the extracts used was insufficient to protect the sperm during the cryopreservation process. Based on the above, it can be assumed that the extraction method used in the present study preserved the antioxidant properties of OFM, satisfactorily improving the post-thawing quality parameters.

The cryopreservation process leads to lipid peroxidation and changes in the proteins involved in the electron transport chain in the mitochondrial membrane. This results in reduced production of ATP, leading to decreased flagellum movements and sperm motility (Meamar et al., 2012; Contino et al., 2023). Therefore, it is expected that the antioxidant cocktail provided by OFM in the freezing extender protects the functionality of cell membranes, including that of mitochondria. These protective effects suggest lower damage to the cell membranes, which could explain the higher viability and motility of spermatozoa observed in treatments containing OFM compared to the Control.

Furthermore, post-thawing sperm motility is influenced by the characteristics of the sperm before freezing (Stintzing and Carle, 2005). Therefore, the calculation of the sperm recovery rate has been used to estimate the success of sperm recovery concerning the characteristics previously shown in the sperm sample (Nicolas et al., 2011; Yeste, 2015; Zhu et al., 2021). In our study, the addition of 6.7% OFM to the freezing extender demonstrated a sperm protective effect, since this treatment improved the recovery rate of total motility and progressive motility up to 3.5 h of incubation.

However, in this study, a slight decrease in the percentage of progressive motility and viability was observed when 10% OFM was added to the freezing extender. This effect could be due to the 10% inclusion level being close to the maximum level of antioxidants that have a beneficial effect against negative ROS effects on sperm cells. In this regard, it has been reported that excessive antioxidants can negatively affect sperm quality (Majzoub and Agarwal, 2020). In a study conducted by Donnelly et al. (1999), the authors reported that supplementing with both ascorbic acid and α-tocopherol has an impact on sperm motility in vitro. The study found that at lower concentrations, these antioxidants protect sperm from ROS, while at higher concentrations, especially when combined, they actually reduce sperm motility. As mentioned previously, OFM contains a high level of antioxidants, including ascorbic acid (El-Mostafa et al., 2014). Furthermore, Majzoub and Agarwal (2020) stated that there is no consensus on the ideal combination, concentration, or type of antioxidants to be added to the freezing extender, and therefore, caution should be exercised as excessive antioxidants may paradoxically damage sperm.

Boar sperm is more susceptible to damage during cooling than other animal species (Parks and Lynch, 1992), which is a result of the high percentage of osmotically inactive water it contains (Du et al., 1994) and the different phospholipid compositions of the plasma membrane of mammalian sperm (Yániz et al., 2015; Zhang et al., 2023). Therefore, a complete routine evaluation of sperm quality should include, in addition to motility, other parameters such as viability, plasma membrane integrity, morphology and acrosome integrity (Eliasson and Treichl, 1971; Gilmore et al., 1996; Přinosilová et al., 2014; Yeste, 2015; Agarwal et al., 2016; Yánez-Ortiz et al., 2022). These tests contribute to a better estimate of the integrity of the plasma membrane and the potential fertility of the sperm (Pérez-Llano et al., 2001; Lechniak et al., 2002). The present study demonstrated the protective effect of OFM on the plasma membranes of the sperm head (eosin-nigrosin test) and tail region (HOST reaction) since sperm frozen in the presence of OFM (3.3, 6.7, or 10.0%) obtained better (p < 0.05) results compared to the Control treatment, even after 3.5 h of post-thawing incubation.

However, it is known that because of cryopreservation, morphological alterations occur that lead to a decrease in fertility (Hancock, 1957; Kruger et al., 1986; García-Herreros et al., 2008). Therefore, morphology assessment has long been used as a routine test to evaluate sperm quality (Hancock, 1957; Pesch and Bergmann, 2006). Moreover, only sperm with intact acrosomes can capacitate and generate an acrosome reaction (Yanagimachi, 2011). On the other hand, the plasma membrane and the acrosomal membrane are the most sensitive parts of the sperm to cryopreservation, with the external acrosomal membrane being more vulnerable than the internal one (Healey, 1969; Pesch and Bergmann, 2006). Amidi et al. (2016) and Bang et al. (2022) indicate that additives with antioxidant properties reduce the impact of damage induced by ROS and cold shock on membranes, preventing alterations to the structure of cryopreserved sperm. Previous studies have consistently demonstrated the beneficial effects of additives with antioxidant properties on cryopreserved spermatozoa from rams (Uysal and Bucak, 2007), bucks (Bucak et al., 2010), boars (Kaeoket and Chanapiwat, 2023), and stallions (Ghallab et al., 2017).

In this study, there were no differences between treatments (p > 0.05) in sperm morphology or acrosome integrity. This suggests that the mucilage components do not by themselves alter the morphology of boar sperm. Despite, the techniques used in this study did not show any visible alterations in the morphology or acrosome structure, other studies have used more precise methods to evaluate these aspects, so these findings will need to be confirmed in future studies.

The *Opuntia* genus plants are found in many regions across America, the Mediterranean, Asia, Africa, and Australia, as they can adapt to different climates and environments (Anderson, 2001). The method utilized in this study for extracting mucilage is straightforward. It involves cleaning the cladode and extracting the mucilage using basic materials that are typically available in most laboratories. In contrast, other authors, such as Sepúlveda et al. (2007), Hussein Banana et al. (2021), and Allai et al. (2023), describe more complex extraction methods that require costly reagents, solvents, or specialized equipment. However, it's important to note that the practical use of OFM may be restricted to regions where it is accessible, similar to other plants with antioxidant properties.

Finally, it is important to note that this study utilized a minimal number of animals, which were previously tested for their freezing capacity and were mostly of similar age and breed. The number of samples used, however, was sufficient to significantly determine the effectiveness of OFM in enhancing the motility and viability of boar sperm during the freezing-thawing process. It's also important to consider that various factors could impact the ability of sperm to freeze, including the time of year, diet, breed, ejaculate fractions, sperm selection, and holding time (Yeste, 2016), in addition to the individual classification of boars and their ejaculates into good, moderate, and poor freezers (Roca et al., 2006). Furthermore, some OFM physicochemical characteristics could be affected by factors such variety, environment, growing area, fertilization, season, maturation stage, and post-harvest procedures (Martins et al., 2023). Therefore, based on the factors mentioned above, it is certain that future research on the use of OFM in the freezing of boar sperm will be guaranteed.

## Conclusion

In agreement with our hypothesis, the OFM inclusion in the lactose-egg yolk freezing extender improves the motility and viability parameters of boar sperm subjected to the freezing-thawing process. The addition of 6.7% OFM in the freezing extender improves motility, motility recovery rate, viability, and HOST reaction of thawed boar spermatozoa. The inclusion of OFM in the freezing extender does not appear to have an effect on sperm morphology or acrosomal integrity. Therefore, OFM can be used as an additive to enhance the quality of cryopreserved boar sperm.

## Data Availability

Research data is only available upon request.
